# Art-based interventions for women’s mental health in pregnancy and postpartum: A meta-analysis of randomised controlled trials

**DOI:** 10.3389/fpsyt.2023.1112951

**Published:** 2023-02-15

**Authors:** Jialu Qian, Shiwen Sun, Man Wang, Xiangyu Sun, Xiaoyan Yu

**Affiliations:** ^1^Faculty of Nursing, School of Medicine, Zhejiang University, Hangzhou, China; ^2^Department of Obstetrics, Women's Hospital School of Medicine, Zhejiang University, Hangzhou, China

**Keywords:** art therapy, psychological well-being, pregnant, postpartum, meta-analysis

## Abstract

**Objective:**

Pregnant and postpartum women are vulnerable to psychological problems with a high estimated prevalence. To date, there is no meta-analysis that specifically assesses the effectiveness of art-based interventions to improve mental health in pregnant and postpartum women. The objective of this meta-analysis was to assess the efficacy of art-based interventions when delivered to pregnant and postpartum women.

**Methods:**

Systematic literature searches were conducted from the inception to 6 March 2022 in seven English databases, including PubMed, Embase, Cochrane Central Register, CINAHL, ProQuest, Scopus, and Web of Science. Randomised controlled trials (RCTs) reporting art-based interventions targeting the improvement of women’s mental health in pregnancy and postpartum were included. Cochrane risk of bias tool was applied to assess evidence quality.

**Results:**

Twenty-one randomised controlled trials (RCTs) involving 2,815 participants were eligible for data analysis. A pooled analysis demonstrated that art-based interventions significantly reduced anxiety (SMD = −0.75, 95% CI = −1.10 to −0.40) and depression symptoms (MD = −0.79, 95% CI = −1.30 to −0.28). However, art-based interventions did not alleviate stress symptoms as expected in our findings. Subgroup analysis demonstrated that intervention implementation time, intervention duration and music selected by the participants vs. not could have influence on the efficacy of art-based intervention for anxiety.

**Conclusion:**

In perinatal mental health, art-based interventions may be effective in alleviating anxiety and depression. In the future, we still need to conduct high-quality RCTs to validate our findings and enrich clinical application of art-based interventions.

## 1. Introduction

The transition to motherhood is an important developmental milestone in many women’s lives (1). Many pregnant women report that they are more aware of judgement from others and experience a burden of complying with very limited understandings of motherhood, which can adversely affect their mental health (2). The postpartum period can be a time of increased vulnerability, as giving birth and adjusting to being a parent for the first time or juggling multiple caregiver roles can be very stressful for mothers (3).

There are various psychological problems that may occur during pregnancy and the postpartum stage, and anxiety, depression and stress are the most prominent (4–6). The COVID-19 pandemic increased anxiety, fear and distress among women in perinatal phase (7). Perinatal anxiety disturbs roughly 20.7% of pregnant and postpartum women (8). Approximately 11.9% of women suffer from depression during pregnancy (9). Up to 84% of women suffer from perinatal stress (10). These psychological issues may have a potential impact on women’s and children’s health (5). Some mental disorders (e.g., postpartum depression) have been reported a higher risk of suicide during the perinatal period (11). Less effective parenting behaviours, reduced maternal sensitivity and decreased ability to breastfeed their babies are closely associated with the severity of women’s psychological problems (12, 13). Adverse child consequences include affected infant and early childhood mental health (14), impaired mother–child interaction (15) and child obesity (16).

Given the high prevalence rates and adverse outcomes of mental health conditions, the need for preventative care is emphasized. Providing care that takes psychological experiences into account may provide childbearing women with more useful assistance (17, 18). Pharmacological treatment is a common strategy adopted for the treatment of psychopathological conditions in pregnant and postpartum women (19); however, it is related to unwanted side effects, drug dependence and transmission of drugs to infants *via* breast milk (20). Therefore, research efforts have been directed towards identifying new initiatives for supporting pregnant and postpartum women.

Art-based intervention, as one of the available nonpharmacological treatments, has no obvious side effects (21). It is inexpensive and demands little time and energy (22). Art-based therapy is a type of psychotherapy that uses art media as its main mode to express and communicate messages (23). Art-based interventions include music, singing, dance movement therapy and so on. They all have a common purpose of stimulating various sensations and creating a safe environment for expressing oneself, being creative, and being imaginative *via* arts (24). Attention restoration theory (25) and the body–mind model (26) can be used to understand the mechanism of art-based interventions. Previous reviews have confirmed the benefits of art-based interventions on mental health (27, 28).

Studies of art-based interventions conducted in pregnant and postpartum women to improve their psychological well-being are accumulating. An integrative review suggests that art-based experiences are beneficial for women in the process of becoming a mother and can be conducive to women’s wellbeing (29). Music therapy has been found to reduce postpartum anxiety (20) and depression (30) in relevant reviews. However, these studies only focused on one specific art therapy. In this field, other types of art-based intervention, such as singing (31) and painting (32), have also been conducted. Art-based intervention was found to be associated with effectively reducing anxiety during labour and pregnancy, reducing postpartum depression and supporting bonding between the mother and infant (6). Nonetheless, some studies have not found significant improvements in psychological well-being (33, 34).

Given the lack of consensus on the efficacy of art-based interventions, a meta-analysis was performed to explore the effects of art-based interventions when delivered to pregnant and postpartum women. We aim to provide evidence-based information regarding the effectiveness of art-based interventions on the prevention of mental health symptoms among pregnant and postpartum women.

## 2. Materials and methods

### 2.1. Design

We followed The Preferred Reporting Items for Systematic Reviews and Meta-Analyses (PRISMA) guidelines (35). Our study protocol was registered on the PROSPERO database (CRD42022316809).

### 2.2. Search strategy

The electronic databases, including PubMed, Embase, Cochrane Central Register, CINAHL, ProQuest, Scopus, and Web of Science, were comprehensively searched by two independent researchers (JLQ and SWS). For grey literature, OpenGrey was also searched. There was no restriction of publication date, and all the studies available from the inception until 6 March 2022 were incorporated. According to the PICOS approach, combinations of keywords, Emtree terms, and subject headings (MeSH terms) were used. A summary of the final search strategies is shown in Supplementary Table 1. Furthermore, the reference lists of the included studies and relevant reviews were examined for potentially eligible studies.

### 2.3. Inclusion and exclusion criteria

PICOS approach was used to identify the inclusion criteria of this study: (1) Participants: healthy pregnant women or puerpera (within the 1st year following childbirth) over the age of 18 with no perinatal complications or mental health diagnoses; (2) Intervention: art-based intervention implemented during pregnancy with the primary or secondary aim of improving women’s mental health. There were no restrictions on intervention settings, frequency, or duration. Possible art-based interventions included but were not limited to: listening to music, singing, dancing or painting; (3) comparison: usual care (routine health care and education), waitlist (participants who will receive intervention after active intervention group) or no interventions; (4) outcomes: no less than one of the psychological outcomes was reported: anxiety or distress or grief or depression or stress or posttraumatic stress disorder measured by self-reported psychological inventories; (5) study design: randomised controlled trials (RCTs); and (6) English-language original articles. These were the exclusion criteria: (1) women who experienced pregnancy-related crises (such as perinatal loss or infertility); (2) studies integrating art-based interventions with other interventions; (3) duplicated publications; and (4) studies without sufficient data.

### 2.4. Study selection

EndNote Version. X9 software was used in order to manage data better. First, after automatic and manual duplication removal, two researchers worked independently on title and abstract screening. Second, full texts were carefully reviewed by two independent researchers to check the identified studies according to the inclusion and exclusion criteria. Third, if any discrepancy appeared, an agreement on the inclusion of studies was reached through the consultation of a third author (MW).

### 2.5. Extraction of data

The data from eligible studies was extracted using a standardised table. Research members discussed and revised the data extraction table after piloting it with a subsample of eligible studies. We extracted data including author, year of publication, country, number of samples, population, details of intervention, control, time points for evaluation, and measurements. Data extraction was carried out independently by two authors (JLQ and SWS). A third reviewer (MW) resolved inconsistencies in data extraction. We attempted to request the data from the corresponding author if the data were insufficient.

### 2.6. Risk of bias in quality assessment

Two reviewers (JLQ and SWS) evaluated the study quality independently by adopting the Cochrane risk of bias tool (36). We assessed bias as a judgement (low risk, unclear, and high risk), and each included study was rated based on its risk of bias (high, moderate, or low quality). Discrepancies were resolved with the help of a third reviewer (MW).

### 2.7. Statistical analyses

Review Manager 5.3 was utilised for the meta-analysis. The effect size was evaluated *via* the changes in mean scores for psychological outcomes from baseline to postintervention. Given that continuous variables were used in this study, if the outcomes were measured adopting the same tool, mean differences (MDs) and 95% confidence intervals (CIs) were used. When the same outcome was assessed by different measurements, standard mean differences (SMDs) and 95% CIs were calculated (37). A SMD value of ≤0.20, =0.50, and ≥ 0.80 was viewed as a small, moderate and large effect size (37). Heterogeneity was evaluated through the χ^2^ test and I^2^ test. A fixed effects model was chosen in the case of the *p* value was >0.1 or I^2^ < 50%. Otherwise, we adopted a random effects model (38). To assess the efficacy of art-based interventions in a variety of trial categories, we also conducted subgroup analyses. A subgroup analysis was conducted when there were more than ten studies in a meta-analysis available for each characteristic modelled (39). Subgroup analyses were based on the intervention implementation time (antenatal or during labour or postnatal), duration of the intervention (a single session vs. multiple session) and music selected by the participants vs. not. We performed Egger’s tests for the assessment of potential publication bias. *Post hoc* sensitivity analyses were conducted (using leave-one-out analysis) to examine the robustness of the results and test the influence of a single trial with a disproportionately large effect.

## 3. Results

### 3.1. Study selection

**Figure 1:**
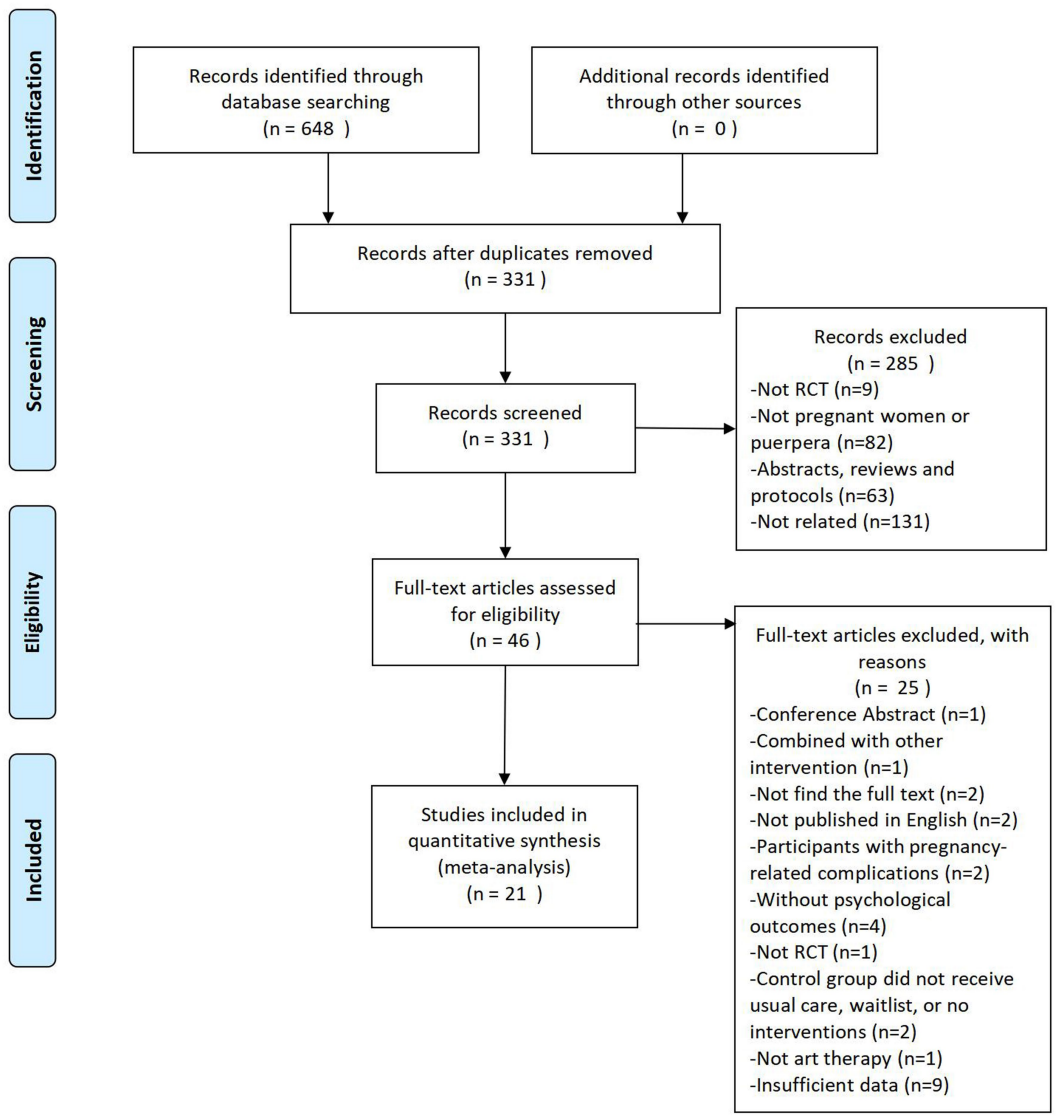
PRISMA flow diagram.

The electronic of 7 databases searches yielded 648 records. After removing 317 duplicates, the remaining 331 articles were screened. Following titles and abstract screening, another 285 articles were excluded. Eligibility was determined by reviewing 46 full-text articles. Twenty-five articles were excluded due to the following reasons: conference abstract (*n* = 1), being combined with other interventions (*n* = 1), not being able to find the full-text articles (*n* = 2), non-English studies (*n* = 2), participants with pregnancy-related complications (*n* = 2), studies without psychological outcomes (*n* = 4), not RCTs (*n* = 1), studies with control groups not receiving usual care, waitlist or no interventions (*n* = 2), studies not involving art-based interventions (*n* = 1) and studies with insufficient data (*n* = 9). Ultimately, 21 studies met the criteria for meta-analysis (31, 33, 34, 40–57). As shown in Figure 1, a PRISMA flow diagram illustrates the process of selecting studies.

### 3.2. Study characteristics

The eligible studies’ characteristics are showed in Supplementary Table 2. Studies were published from 2005 to 2022. There were seven studies in Turkey, seven in China, three in Germany, two in Iran, and one each in Italy and Romania. The number of participants in each trial varied from 30 to 409 participants, with a total of 2,815 participants included in the review. For intervention type, most of included studies adopted music therapy, while 2 studies used singing therapy. For the intervention implementation time, six studies were conducted in the antenatal period, 11 studies were conducted during delivery, and the remaining four studies were conducted in the postnatal period. Studies measuring posttraumatic stress disorder or distress or grief were not found in the search and therefore this meta-analysis focused exclusively on anxiety, depression, and stress as outcome variables. These trials used the State–Trait-Anxiety Inventory (STAI), Visual Analogue Scale (VAS) and Self-Rating Anxiety Scale (SAS) to evaluate anxiety; the Edinburgh Postnatal Depression Scale (EPDS) was adopted to assess the level of depression; and the Perceived Stress Scale (PSS) was utilised to evaluate the severity of stress.

### 3.3. Risk of bias assessment

**Figure 2:**
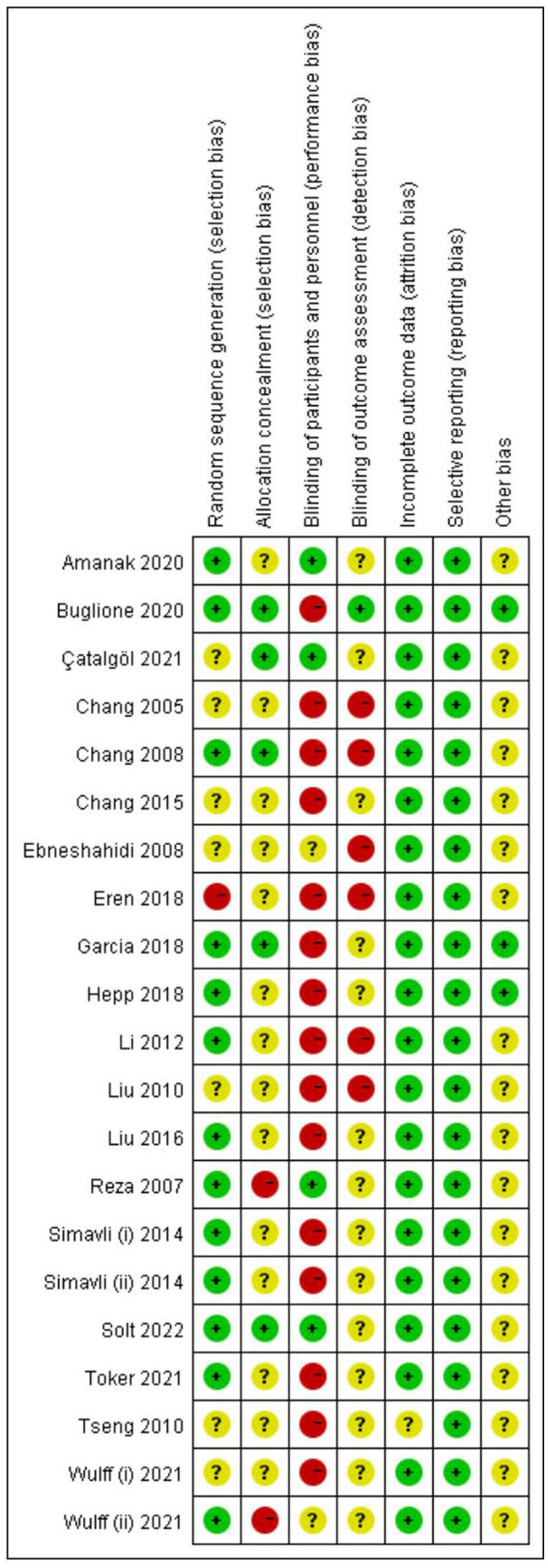
Risk of bias for individual RCTs.

**Figure 3:**
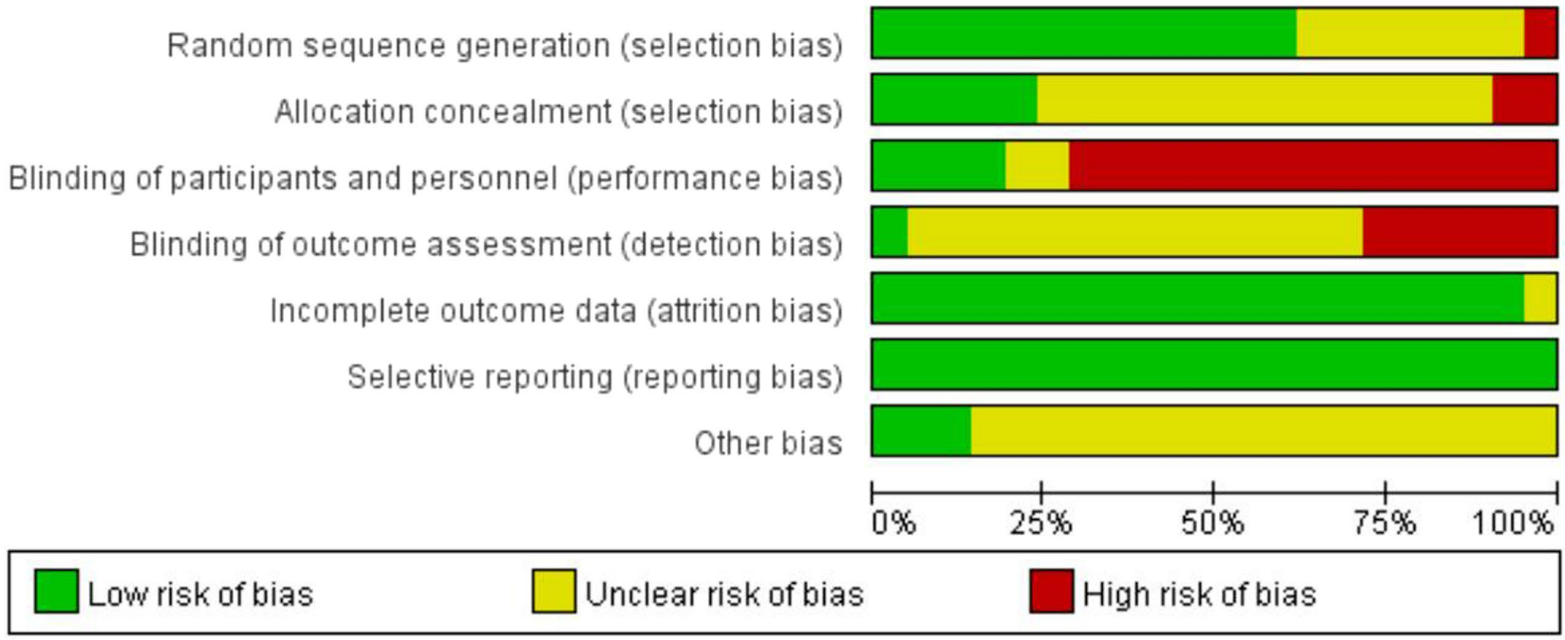
Risk of bias summaries for the included RCTs.

Figures 2, 3 show quality assessments of the 21 included studies. Randomisation was reported in all studies; however, the randomisation method was not described in detail in eight articles. Allocation concealment was sufficiently described in only five studies. Four studies were at a low risk of performance bias. Considering the characteristics of this type of intervention, blinding is difficult, so most studies are at high risk of performance bias. Only one study offered an adequate explanation for blinding the outcome assessments. There were 20 studies with clear evidence of incomplete outcome data. Among the included studies, no selection bias was found.

### 3.4. Intervention effects

#### 3.4.1. Primary outcome: Anxiety symptoms

**Figure 4:**
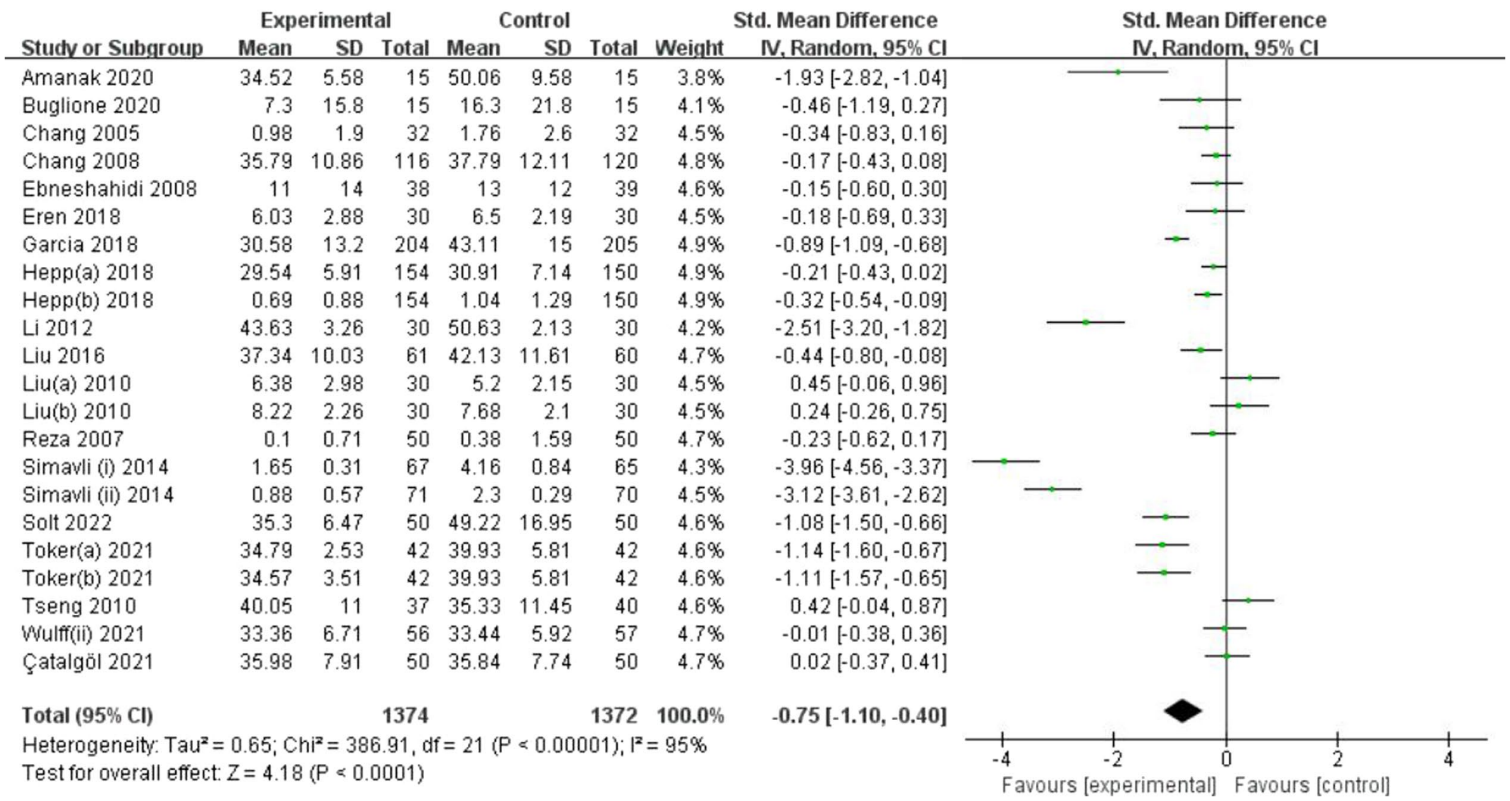
Forest plot of posttreatment anxiety outcome for comparing art-based intervention with control.

Figure 4 shows the impact of art-based interventions on anxiety symptoms. Nineteen trials showed posttreatment anxiety, and we found that the two groups differed significantly. A random-effects model was adopted since the heterogeneity was obvious (I^2^ = 95%, *p* < 0.00001). The SMD was −0.75 (95% CI = −1.10 to −0.40, *p* < 0.0001), suggesting that anxiety symptoms were reduced effectively by art-based interventions.

#### 3.4.2. Secondary outcome: Depression symptoms

**Figure 5:**
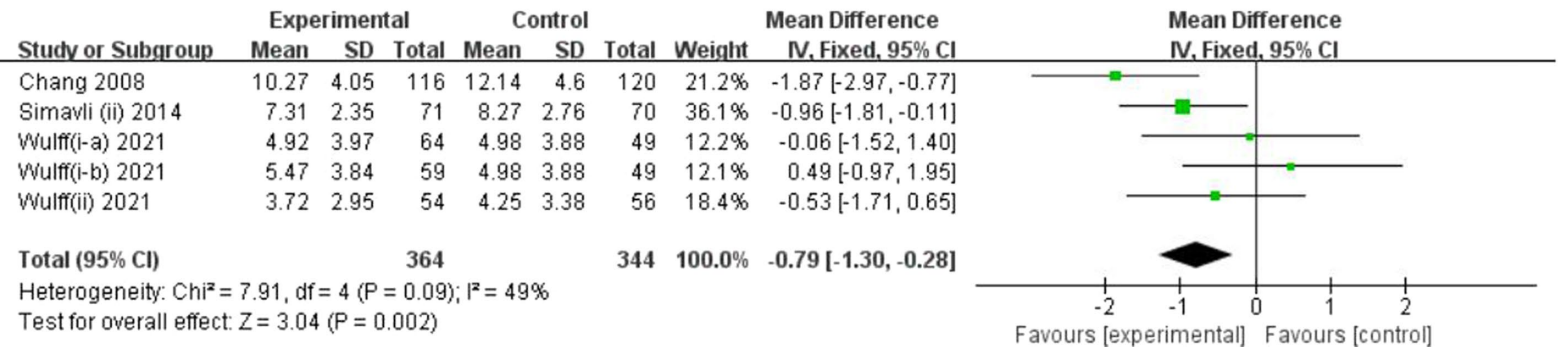
Forest plot of posttreatment depression outcome for comparing art-based intervention with control.

Figure 5 displays the results of art-based interventions on depression symptoms. A meta-analysis that included four studies demonstrated a significant effect of art-based interventions on depression symptom reduction. A fixed-effects model was chosen due to insignificant heterogeneity (I^2^ = 49%, *p* = 0.09). The MD was −0.79 (95% CI = −1.30 to −0.28, *p* = 0.002).

#### 3.4.3. Secondary outcome: Stress symptoms

**Figure 6:**
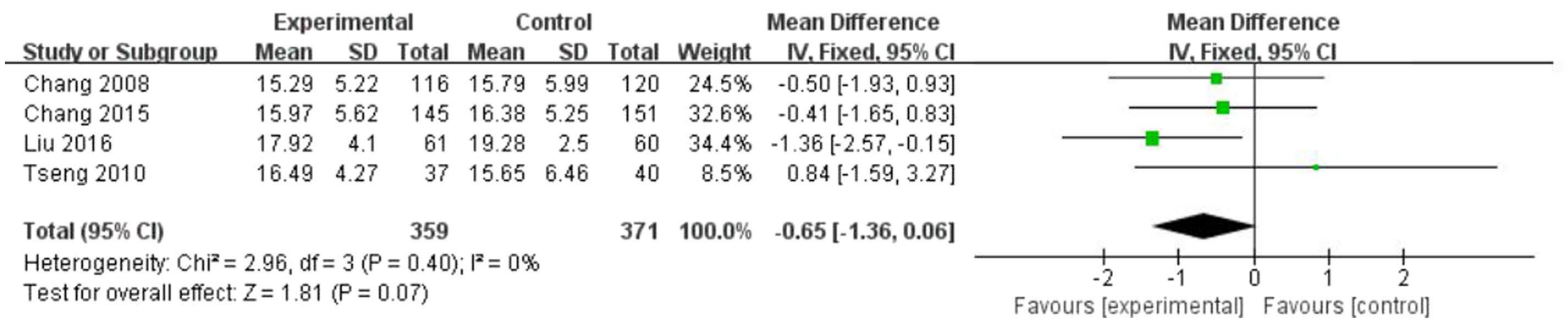
Forest plot of posttreatment stress outcome for comparing art-based intervention with control.

Figure 6 illustrates the results of art-based interventions on stress symptoms. According to a meta-analysis including four studies, there was no significant effect on stress reduction. Data analysis revealed no significant heterogeneity (I^2^ = 0%, *p* = 0.40). Therefore, we used a fixed-effects model. The MD was −0.65 (95% CI = −1.36 to 0.06, *p* = 0.07).

### 3.5. Subgroup analysis

Analysis of subgroups was conducted to determine whether the effect sizes varied based on the intervention implementation time (antenatal or during labour or postnatal), intervention duration (single session vs. multiple session) and music selected by the participants vs. not for the primary outcome anxiety. See Supplementary Figures 1–3.

We found a significant difference between different intervention implementation times (antenatal or during labour or postnatal) for anxiety. Art-based interventions conducted during labour appeared to be effective in decreasing anxiety (SMD = −1.03, 95% CI = −1.64 to −0.42, *p* = 0.0009). However, no comparative efficiency of the art-based interventions conducted in the antenatal period (SMD = −0.38, 95% CI = −0.82 to 0.05, *p* = 0.08) or the postnatal period (SMD = −0.39, 95% CI = −0.98 to 0.19, *p* = 0.19) was found. Additionally, we carried out a subgroup analysis according to the intervention duration (a single session vs. multiple sessions) for anxiety. Studies using a single session (SMD = −0.90, 95% CI = −1.48 to −0.31, *p* = 0.003) resulted in better results than using multiple sessions (SMD = −0.53, 95% CI = −0.90 to −0.16, *p* = 0.005) in relieving anxiety symptoms. The subgroup analysis also showed that music selected by the participants had a better intervention efficacy (SMD = −1.19, 95% CI = −2.08 to −0.30, *p* = 0.009) compared with music not selected by participants (SMD = −0.36, 95% CI = −0.57 to −0.15, *p* = 0.0009).

### 3.6. Publication bias

Egger’s linear regression test was adopted to examine potential publication bias. Egger’s test did not indicate publication bias for anxiety (*t* = −1.42, *p* = 0.170), depression (*t* = 2.74, *p* = 0.071) or stress (*t* = −0.01, *p* = 0.996).

### 3.7. Sensitivity analysis

With the purpose of identifying the sources of the high heterogeneity in anxiety, sensitivity analyses were performed. However, the results of sensitivity analyses showed that heterogeneity was not obviously reduced after excluding any of the included studies. The sensitivity analysis is shown in Supplementary Table 3.

## 4. Discussion

### 4.1. Main findings

Studies in the past have found inconsistent findings in regard to the effectiveness of art-based interventions, to our knowledge, it is the first meta-analysis to examine the effect of art-based interventions on pregnant and postpartum women’s mental health. According to this meta-analysis, significant differences were observed between the art-based intervention group and the control group in anxiety and depression at postintervention. Art-based interventions were found to be effective for relieving anxiety and depression in our meta-analysis. Stress reduction, however, was insignificantly different between the two groups. Subgroup analyses showed that the implementation time, intervention duration, and music selected by participants versus those who did not had a significant impact on the effects of the art-based interventions. There was no evidence of publication bias in anxiety, depression or stress according to Egger’s test. The sensitivity analyses did not find the causes of the significant heterogeneity for anxiety. The heterogeneity may have resulted from the different measurements and intervention types.

### 4.2. Comparisons with other studies

A previous systematic review and meta-analysis showed that art therapy effectively reduces anxiety and depression symptoms in an unselected population (58). In the present study, art-based interventions showed a prominent influence on reducing anxiety and depression symptoms. The results were consistent with those of previous studies. The Bodymind model reported that art therapy could activate bodymind processes, which facilitate the process of activating, reorganising, growing, and reintegrating (26). Art-based interventions could promote emotional regulation (59) and have benefits on psychological symptoms (60). A number of studies have described art-based interventions as psychological interventions resulting in fewer anxiety and depression symptoms (61, 62). The current study confirmed these conclusions regarding the possible ability of art-based interventions to alleviate anxiety and depression in pregnant and postpartum women. However, we did not observe a significant effect of art-based interventions for stress. Similarly, a meta-analysis conducted in patients with breast and gynaecological cancers (63) reported that stress symptoms were not significantly reduced by art therapy. It is worth noting that a previous meta-analysis only included two studies, and the current meta-analysis only involved four studies for stress analysis. The number of included studies for stress analysis was small. The effect of art-based interventions on stress needs to be verified by further research. Studies (64, 65) suggested that mindfulness-based art therapy provided more proactive and direct benefits than art-making alone. Therefore, whether art-based interventions combined with mindfulness could have a better intervention efficacy on stress reduction still needs to be studied.

According to the findings of the subgroup analyses, art-based intervention’ effectiveness may be affected by intervention implementation time, intervention duration, and the music chosen by participants or not. With respect to the intervention implementation time, the art-based intervention was only effective for anxiety during labour. Anxiety was significantly correlated with pain and fatigue throughout the process of labour (66), and it might lead to obvious anxiety symptoms during delivery. Subgroup analysis of intervention duration also showed that studies using a single session appeared to be more effective than using multiple sessions in relieving anxiety symptoms. It could be because single session art-based interventions were more likely to be implemented during delivery, a time in which anxiety symptoms are particularly pronounced. Therefore, art-based therapy showed good results in decreasing severe anxiety during labour (67, 68). Nevertheless, art-based interventions were ineffective in the antenatal period and postnatal period in our findings. According to a meta-analysis, with increasing maternal age, music interventions reduce prenatal anxiety in a nonsignificant manner (69). Additionally, there were three unique trajectories of postpartum anxiety among women, and different combinations of risk factors may result in different responses to interventions among these groups of women (70). In this study, we did not strictly limit maternal age and distinguish subtypes of anxiety of the included population, which may influence the intervention effects on women. The subgroup analysis showed that music selected by the participants had a better intervention efficacy on anxiety compared with music not selected by participants. It is suggested that art-based intervention should take participant’s preference into consideration, so that it could achieve a better therapeutic effect.

### 4.3. Strengths and limitations

It is the first meta-analysis to assess the effects of art-based interventions on women’s mental health in pregnancy and postpartum with a wide range of electronic databases. Two researchers independently searched databases, selected studies, extracted data and assessed study quality, which ensured the rigour of this study. Additionally, given its high prevalence and severe consequences of various psychological problems across pregnancy and the postpartum period, the results of the present study could possess a positive effect on the management of perinatal mental health.

Some limitations also need to be mentioned. Firstly, we only included studies published in English. This may cause language bias (71), which may have led to an overestimation of effects because an English-language publication is more likely to have positive findings (72). Secondly, most included studies had a moderate quality; therefore, it is important to interpret the results of this study with caution. Thirdly, heterogeneity was noted in the meta-analysis for anxiety. We could not adequately explain the sources of heterogeneity, although subgroup and sensitivity analyses were further performed.

### 4.4. Clinical implications and future research

Considering the positive results of art-based interventions for anxiety and depression in pregnant and postpartum women, this kind of intervention could be applied in the management of perinatal mental health. Art-based interventions seemed to be interesting, safe and cost-effective for women (73). Art-based interventions could provide a new avenue for preventing perinatal mental health problems and help to reduce the likelihood of developing a mood disorder and needing pharmacological treatment. Women with greater symptom severity may need to receive further psychotherapy or psychopharmacological treatments. In regard to the negative effects of art-based interventions for stress and antenatal and postnatal anxiety, on the one hand, future studies could consider combining art therapy with mindfulness (64, 74, 75) or cognitive behavioural treatment (76) to improve the intervention efficacy; on the other hand, future studies could recruit participants based on maternal age or characteristics of their psychological problems (e.g., severity, trajectories) to understand the efficacy of art-based interventions among populations with different characteristics. In our study, we only included RCTs implementing music and singing therapy. In the future, more high-quality RCT studies applying other types of art therapy (e.g., painting, dancing) are needed to investigate whether different intervention types may lead to different efficacy and enrich the clinical application of art-based interventions.

## 5. Conclusion

Overall, the results of this study indicate that art-based intervention was an effective psychotherapy for pregnant or postpartum women to alleviate anxiety and depression, but not for stress relief. Art-based intervention was effective for anxiety during labour rather than the antenatal period and postnatal period. Other psychotherapies, such as mindfulness and cognitive behavioural treatment, could be combined with art-based interventions to enhance efficacy. The clinical features and psychological characteristics of women need to be considered in future research. These findings call for the development of high-quality RCTs in the future to confirm the current results and to facilitate the dissemination of art-based interventions.

## Author contributions

JQ and XY: study design. JQ, SS and WM: data collection. JQ, SS, WM and XS: data analysis. XY: study supervision. JQ: manuscript writing. JQ, SS, WM, XS and XY: critical revisions for important intellectual content. All authors contributed to the article and approved the submitted version.

## Funding

This study was funded by the Zhejiang Medical and Health Research Project (Foundation number: 2022KY859) and China Scholarship Council (grant number 202106320274).

## Conflict of interest

The authors declare that the research was conducted in the absence of any commercial or financial relationships that could be construed as a potential conflict of interest.

## Publisher’s note

All claims expressed in this article are solely those of the authors and do not necessarily represent those of their affiliated organizations, or those of the publisher, the editors and the reviewers. Any product that may be evaluated in this article, or claim that may be made by its manufacturer, is not guaranteed or endorsed by the publisher.

## Supplementary material

The Supplementary material for this article can be found online at: https://www.frontiersin.org/articles/10.3389/fpsyt.2023.1112951/full#supplementary-material
