# Supplementary material for: Art-based interventions for women’s mental health in pregnancy and postpartum: A meta-analysis of randomised controlled trials
Source: Front Psychiatry. 2023 Feb 15;14:1112951. doi: 10.3389/fpsyt.2023.1112951 (PMC9976780; doi:10.3389/fpsyt.2023.1112951)
Supplement: Supplementary file 1 [file Table_1.DOCX]

**Supplementary Table 1：Final search strategies of each database**

| Database | Search Strategy | Literature |
| --- | --- | --- |
| Pubmed | 1. pregnancy[MeSH Terms] 2. pregnant women[MeSH Terms] 3. delivery[Title/Abstract] 4. childbirth[MeSH Terms] 5. puerpera[Title/Abstract] 6. postpartum[Title/Abstract] 7. postnatal[Title/Abstract] 8. puerperium[MeSH Terms] 9. maternal[Title/Abstract] 10. OR/ 1-9 11. art therapy[MeSH Terms] 12. singing[Title/Abstract] OR dancing[Title/Abstract] OR painting[Title/Abstract] OR drawing[Title/Abstract] OR calligraphy[Title/Abstract] OR music[Title/Abstract] OR sculpture[Title/Abstract] OR collage[Title/Abstract] OR poetry[Title/Abstract] OR drama[Title/Abstract] OR clay[Title/Abstract] 13. OR/ 11-12 14. mental health[Title/Abstract] 15. mental illness[Title/Abstract] 16. mental disorder[MeSH Terms] 17. depression[Title/Abstract] 18. anxiety[Title/Abstract] 19. stress[Title/Abstract] 20. posttraumatic stress disorder[MeSH Terms] 21. distress[Title/Abstract] 22. grief[MeSH Terms] 23. fear of childbirth[Title/Abstract] 24. depressive symptoms[MeSH Terms] 25. negative emotions[Title/Abstract] 26. OR/ 14-25 27. randomized controlled trial[Publication Type] 28. randomized[Title/Abstract] 29. OR/ 27-28 30. 10 AND 13 AND 26 AND 29 31. Article Type: Randomized Controlled Trial | 88 |
| Embase | 1. 'pregnancy'/exp 2. 'pregnant woman'/exp 3. 'obstetric delivery'/exp 4. 'childbirth'/exp 5. 'puerperium'/exp 6. 'maternal'/exp 7. 'puerpera' OR 'postpartum'/exp OR 'postpartum' OR 'postnatal':ab 8. OR/ 1-7 9. 'art therapy'/exp 10. 'singing'/exp 11. 'dancing'/exp 12. 'painting'/exp 13. 'drawing'/exp 14. 'music therapy'/exp 15. 'art'/exp 16. 'poetry therapy'/exp 17. 'clay'/exp 18. 'calligraphy'/exp OR 'calligraphy' OR 'sculpture'/exp OR 'sculpture' OR 'collage' OR 'drama':ab 19. OR/ 9-18 20. 'mental health'/exp 21. 'mental disease'/exp 22. 'depression'/exp 23. 'anxiety'/exp 24. 'posttraumatic stress disorder'/exp 25. 'grief'/exp 26. 'fear of childbirth'/exp 27. 'depressive symptoms'/exp 28. 'mental illness'/exp OR 'mental illness' OR 'mental disorder'/exp OR 'mental disorder' OR 'stress'/exp OR 'stress' OR 'distress'/exp OR 'distress' OR 'negative emotions':ab 29. OR/ 20-28 30. 'randomized controlled trial'/exp 31. 'randomized':ab 32. OR/30-21 33. 8 ADN 19 AND 29 AND 32 | 71 |
| Cochrane Library | 1 MeSH descriptor: [Pregnancy] explode all trees  2 MeSH descriptor: [Pregnant Women] explode all trees  3 MeSH descriptor: [Parturition] explode all trees  4 MeSH descriptor: [Postpartum Period] explode all trees  5 MeSH descriptor: [Postpartum Period] explode all trees  6 (delivery OR puerpera OR postnatal OR maternal):ti,ab,kw (Word variations have been searched)  7 OR/ 1-6  8 MeSH descriptor: [Art Therapy] explode all trees  9 MeSH descriptor: [Singing] explode all trees  10 MeSH descriptor: [Dancing] explode all trees  11 MeSH descriptor: [Paintings] explode all trees  12 MeSH descriptor: [Drawing] explode all trees  13 MeSH descriptor: [Music Therapy] explode all trees  14 MeSH descriptor: [Sculpture] explode all trees  15 MeSH descriptor: [Drama] explode all trees  16 MeSH descriptor: [Clay] explode all trees  17 (calligraphy OR collage OR poetry):ti,ab,kw (Word variations have been searched)  18 OR/ 8-17  19 MeSH descriptor: [Mental Health] explode all trees  20 MeSH descriptor: [Mental Disorders] explode all trees  21 MeSH descriptor: [Depression] explode all trees  22 MeSH descriptor: [Anxiety] explode all trees  23 MeSH descriptor: [Stress Disorders, Post-Traumatic] explode all trees  24 MeSH descriptor: [Psychological Distress] explode all trees  25 MeSH descriptor: [Grief] explode all trees  26 MeSH descriptor: [Depression] explode all trees  27 (mental illness OR stress OR fear of childbirth OR negative emotions):ti,ab,kw (Word variations have been searched)  28 OR/ 19-27  29 MeSH descriptor: [Randomized Controlled Trial] explode all trees  30 (randomized):ti,ab,kw (Word variations have been searched)  31 OR/29-30  32 7 AND 18 AND 28 AND 31 | 64 |
| CINAHL | 1.(pregnancy OR pregnant OR delivery OR childbirth OR puerpera OR postpartum OR postnatal OR puerperium OR maternal)[Abstact]  2.(art therapy OR singing OR dancing OR painting OR drawing OR calligraphy OR music OR sculpture OR collage OR poetry OR drama OR clay)[Abstact]  3.(mental health OR mental illness OR mental disorder OR depression OR anxiety OR stress OR posttraumatic stress disorder OR distress OR grief OR fear of childbirth OR depressive symptoms OR negative emotions)[Abstact]  4.(randomized controlled trial or RCT or randomised control trial)[Abstact]  5.1 AND 2 AND 3 AND 4 | 36 |
| PsychINFO | 1.(pregnancy OR pregnant OR delivery OR childbirth OR puerpera OR postpartum OR postnatal OR puerperium OR maternal)[Abstact]  2.(art therapy OR singing OR dancing OR painting OR drawing OR calligraphy OR music OR sculpture OR collage OR poetry OR drama OR clay)[Abstact]  3.(mental health OR mental illness OR mental disorder OR depression OR anxiety OR stress OR posttraumatic stress disorder OR distress OR grief OR fear of childbirth OR depressive symptoms OR negative emotions)[Abstact]  4.(randomized controlled trial or RCT or randomised control trial)[Abstact]  5.1 AND 2 AND 3 AND 4 | 16 |
| Proquest | 1.(pregnancy OR pregnant OR delivery OR childbirth OR puerpera OR postpartum OR postnatal OR puerperium OR maternal)[Abstact]  2.(art therapy OR singing OR dancing OR painting OR drawing OR calligraphy OR music OR sculpture OR collage OR poetry OR drama OR clay)[Abstact]  3.(mental health OR mental illness OR mental disorder OR depression OR anxiety OR stress OR posttraumatic stress disorder OR distress OR grief OR fear of childbirth OR depressive symptoms OR negative emotions)[Abstact]  4.(randomized controlled trial or RCT or randomised control trial)[Abstact]  5.1 AND 2 AND 3 AND 4 | 63 |
| Scopus | 1.(pregnancy OR pregnant OR delivery OR childbirth OR puerpera OR postpartum OR postnatal OR puerperium OR maternal)[Abstact]  2.(art therapy OR singing OR dancing OR painting OR drawing OR calligraphy OR music OR sculpture OR collage OR poetry OR drama OR clay)[Abstact]  3.(mental health OR mental illness OR mental disorder OR depression OR anxiety OR stress OR posttraumatic stress disorder OR distress OR grief OR fear of childbirth OR depressive symptoms OR negative emotions)[Abstact]  4.(randomized controlled trial or RCT or randomised control trial)[Abstact]  5.1 AND 2 AND 3 AND 4  6.Publication Type: Article | 146 |
| Web of Science | 1. pregnancy OR pregnant OR delivery OR childbirth OR puerpera OR postpartum OR postnatal OR puerperium OR maternal  2. art therapy OR singing OR dancing OR painting OR drawing OR calligraphy OR music OR sculpture OR collage OR poetry OR drama OR clay  3. mental health OR mental illness OR mental disorder OR depression OR anxiety OR stress OR posttraumatic stress disorder OR distress OR grief OR fear of childbirth OR depressive symptoms OR negative emotions  4. randomized controlled trial or RCT or randomised control trial  5. 1 AND 2 AND 3 AND 4  Publication Type: Clinical trial | 164 |
| OpenGrey | Art therapy for mental health | 1 |
