# Supplementary material for: Art-based interventions for women’s mental health in pregnancy and postpartum: A meta-analysis of randomised controlled trials
Source: Front Psychiatry. 2023 Feb 15;14:1112951. doi: 10.3389/fpsyt.2023.1112951 (PMC9976780; doi:10.3389/fpsyt.2023.1112951)
Supplement: Supplementary file 2 [file Table_2.DOCX]

**Supplementary Table 2** Characteristics of the included articles (n=21)

| **Study**  **(year)** | **Country** | **Sample size (intervention/control)** | **Population** | **Intervention type** | **Intervention details** | **Control** | **Evaluation time points** | **Measurements** |
| --- | --- | --- | --- | --- | --- | --- | --- | --- |
| Amanak  (2020) | Turkey | 15/15 | Primiparous pregnant women who had no history of miscarriage or abortion, had a single foetus | Music therapy | Participants were exposed to 30 minutes of instrumental ney music played in the modal rhythm of Segah in the active phase of labour. The same pattern was repeated 3 times, with each round lasting 30 minutes, followed by 30 minutes of intermission. | Usual care | Before and after the intervention | STAI |
| Buglione et al.  (2020) | Italy | 15/15 | Nulliparous women with singleton pregnancies and vertex presentation | Music therapy | Women in the intervention group were offered music in labour, defined as listening to music from the randomization until the delivery of the baby | Usual care | Baseline; 1 h, 24 h, and 48 h postpartum | VAS for anxiety |
| Çatalgöl et al.  (2021) | Turkey | 50/50 | Primiparous women at the 36th gestational week who were directed to Nonstress Test application | Music therapy | Women listened to 12 instrumental Classical Turkish Maqam Music at home during pregnancy and labour | Usual care | Before and after the intervention | STAI |
| Chang et al.  (2015) | China (Taiwan) | 145/151 | Pregnant women at gestational age≥17 weeks | Music therapy | Participants were given the prerecorded CD and asked to listen to the music at least 30 min a day for 2 weeks | Usual care | Baseline; 2 weeks | PSS |
| Chang et al.  (2008) | China (Taiwan) | 116/120 | Pregnant women at gestational age of 18-22 weeks or 30-34 weeks | Music therapy | Participants were given the prerecorded CD and asked to listen to at least one disc (30 minutes) a day for two weeks | Usual care | Baseline; 2 weeks | PSS, STAI, EPDS |
| Chang et al.  (2005) | China (Taiwan) | 32/32 | Women scheduled to receive a caesarean section | Music therapy | Participants listened to music for at least 30 minutes from the start of anaesthesia till the end of surgery | Usual care | Baseline, upon the end of maternal contact, and after completion of the skin suture for the caesarean section | VAS for anxiety |
| Ebneshahidi et al.  (2008) | Iran | 38/39 | Pregnant women aged 18-36 year, scheduled to undergo general anaesthesia and elective caesarean section surgery | Music therapy | Music was administered 15 minutes after arrival at the recovery room for 30 minutes via soft open-air headphones and a tape player | Usual care | Before and after the intervention | VAS for anxiety |
| Eren et al.  (2018) | Turkey | 30/30 | Pregnant women undergoing multiple caesarean section | Music therapy | Songs prepared preoperatively were played at each patient’s desired volume throughout the operation using a stereo player | Usual care | Before and after the intervention | VAS for anxiety |
| Garcia et al.  (2018) | Spain | 204/205 | Nulliparous women coming for routine prenatal care | Music therapy | 40 min/session (with the seven songs on the CD). Listened to the music for 14 sessions, three times a week and at the same time of day | Usual care | Before and after the intervention | STAI |
| Hepp et al.  (2018) | Germany | 154/150 | Women undergoing caesarean delivery | Music therapy | Participants listened to music after entering the operating theatre until skin suture | Usual care | At admission, skin suture, 2 h post-surgery | STAI, VAS for anxiety |
| Li et al.  (2012) | China | 30/30 | Women undergoing elective caesarean delivery | Music therapy | On the day of surgery the participants selected pieces of Chinese classical music and listened to the slow-rhythm music for 30 minutes before undergoing surgery. | Usual care | Before and after the intervention | SAS |
| Liu et al.  (2010) | China (Taiwan) | 30/30 | Primiparas expected to have a normal spontaneous delivery | Music therapy | Participants listened to music for at least 30 minutes during the latent phase and active phase of labour | Usual care | Latent phase and active phase | VAS for anxiety |
| Liu et al.  (2016) | China (Taiwan) | 61/60 | Pregnant women at 18-34 weeks of gestation and had a score on the PSQI score＞5 | Music therapy | Participants listened to music and kept a two-week daily journal stating relevant information of music | Usual care | Baseline; 2 weeks | PSS, STAI |
| Reza et al.  (2007) | Iran | 50/50 | Patients who were scheduled for an elective caesarean section under general anaesthesia | Music therapy | Women listened to CD-player which was started immediately after induction of anaesthesia and continued to the time of wound dressing | Usual care | 0.5 h, 1 h, 2 h, 4 h, 6 h after postanaesthesia care | VAS for anxiety |
| Simavli (i) et al.  (2014) | Turkey | 67/65 | Primiparous women at 37-41 weeks of gestation with singleton pregnancies and babies of cephalic presentation | Music therapy | The music was played all the time with a 20-min break for every hour of music from 2 cm cervical dilatation to the third stage | Usual care | Baseline; latent phase; active phase; second stage; 2 h postpartum | VAS for anxiety |
| Simavli (ii) et al.  (2014) | Turkey | 71/70 | Primiparous women with a 37-41 weeks of gestation | Music therapy | During the labour, the melodies previously selected by the pregnant women were played all the time with a 20-min break for every two hours of music and music was continued to the end of the third stage | Usual care | 1, 4, 8, 16, 24 h in the postpartum period for anxiety;Baseline, postpartum day one and day eight for depression | VAS for anxiety, EPDS |
| Solt et al.  (2022) | Turkey | 50/50 | Pregnant women who applied to private hospital for vaginal and caesarean delivery | Music therapy | Women listened to music of their preference between the beginning and the end of the episiotomy repair (15-20 min) in the delivery room | Usual care | Before and after the intervention | STAI |
| Toker et al.  (2021) | Turkey | 42/42/42 | Mothers who had a live birth and a healthy newborn | Music therapy | On the first day after the C-section at baseline, intervention Groups 1 and 2 listened to music for 30 minutes once a day and twice a day, respectively | Usual care | Baseline; Day 1 and Day 2 after the intervention | STAI |
| Tseng et al.  (2010) | China (Taiwan) | 37/40 | Postnatal women who delivered a mature and normal newborn | Music therapy | Women listened to music at home for at least 30 minutes a day over two weeks. | Usual care | Baseline; 2 weeks | PSS, STAI |
| Wulff (i) et al.  (2021) | Germany | 64/59/49 | Women aged above 18 years, without serious comorbidities or pregnancy risks | Music therapy/ Singing therapy | Participants were asked to listen and relax to music/to sing children’s songs and lullabies at home on a daily basis for at least 10-15 min/day until the time of birth | Usual care | Baseline; 6 weeks | EPDS |
| Wulff (ii) et al.  (2021) | Germany | 59/61 | New mothers within 48 h after childbirth, aged above 18 years, without serious comorbidities or pregnancy risks | Singing therapy | The intervention session took place every second week. Women participated between 1~3 times. Participants practised 45 min during the intervention session. They were asked to implement the intervention daily at home. | Usual care | Baseline; 10 weeks | STAI, EPDS |

Abbreviations: STAI= State-Trait Anxiety Inventory, SAS= Self-Rating Anxiety Scale, EPDS=Edinburgh Postnatal Depression Scale, PSS= Perceived Stress Scale, VAS= Visual Analogue Scale, PSQI=Pittsburgh Sleep Quality Index.
