# Supplementary material for: Art-based interventions for women’s mental health in pregnancy and postpartum: A meta-analysis of randomised controlled trials
Source: Front Psychiatry. 2023 Feb 15;14:1112951. doi: 10.3389/fpsyt.2023.1112951 (PMC9976780; doi:10.3389/fpsyt.2023.1112951)
Supplement: Supplementary file 3 [file Table_3.DOCX]

**Supplementary Table 3. Sensitivity analyses for anxiety**

| **Anxiety severity (at post assessment)** | | | |  |
| --- | --- | --- | --- | --- |
| **Study omitted** | **I^2^** | **SMD[95% CI]** | **P-value** | |
| Amanak (2020) | 95% | -0.70 [-1.05,-0.35] | 0.0001 | |
| Buglione (2020) | 95% | -0.76 [-1.12,-0.40] | <0.0001 | |
| Çatalgöl (2021) | 95% | -0.79 [-1.15,-0.42] | <0.0001 | |
| Chang (2005) | 95% | -0.77 [-1.13,-0.41] | <0.0001 | |
| Chang (2008) | 95% | -0.78 [-1.16,-0.41] | <0.0001 | |
| Ebneshahidi (2008) | 95% | -0.78 [-1.14,-0.41] | <0.0001 | |
| Eren (2018) | 95% | -0.78 [-1.14,-0.41] | <0.0001 | |
| Garcia (2018) | 95% | -0.75 [-1.13,-0.36] | 0.0001 | |
| Hepp (2018) | 95% | -0.81 [-1.22,-0.39] | 0.0001 | |
| Li (2012) | 94% | -0.67 [-1.02,-0.32] | 0.0001 | |
| Liu (2010) | 95% | -0.86 [-1.22,-0.49] | <0.00001 | |
| Liu (2016) | 95% | -0.77 [-1.14,-0.40] | <0.0001 | |
| Reza (2007) | 95% | -0.78 [-1.14,-0.41] | <0.0001 | |
| Simavli (i) (2014) | 92% | -0.60 [-0.89,-0.30] | <0.0001 | |
| Simavli (ii) (2014) | 93% | -0.63 [-0.94,-0.32] | <0.0001 | |
| Solt (2022) | 95% | -0.73 [-1.10,-0.37] | <0.0001 | |
| Toker (2021) | 95% | -0.71 [-1.09,-0.34] | 0.0002 | |
| Tseng (2010) | 95% | -0.81 [-1.16,-0.45] | <0.0001 | |
| Wulff(ii) (2021) | 95% | -0.79 [-1.15,-0.42] | <0.0001 | |
