# Supplementary figures and images for: Art-based interventions for women’s mental health in pregnancy and postpartum: A meta-analysis of randomised controlled trials
Source: Front Psychiatry. 2023 Feb 15;14:1112951. doi: 10.3389/fpsyt.2023.1112951 (PMC9976780; doi:10.3389/fpsyt.2023.1112951)

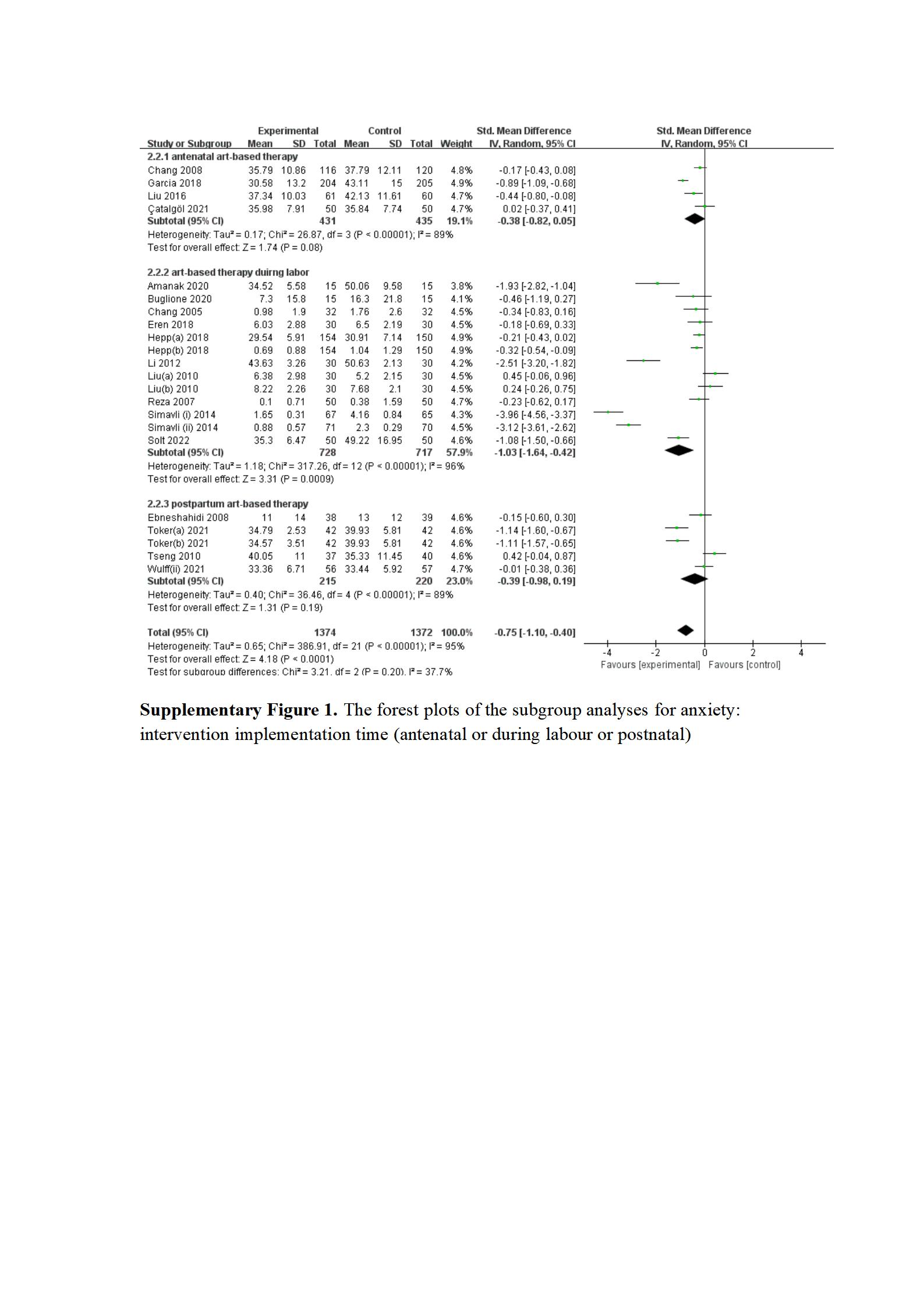

Supplement: Supplementary file 4 [file Image_1.JPEG]

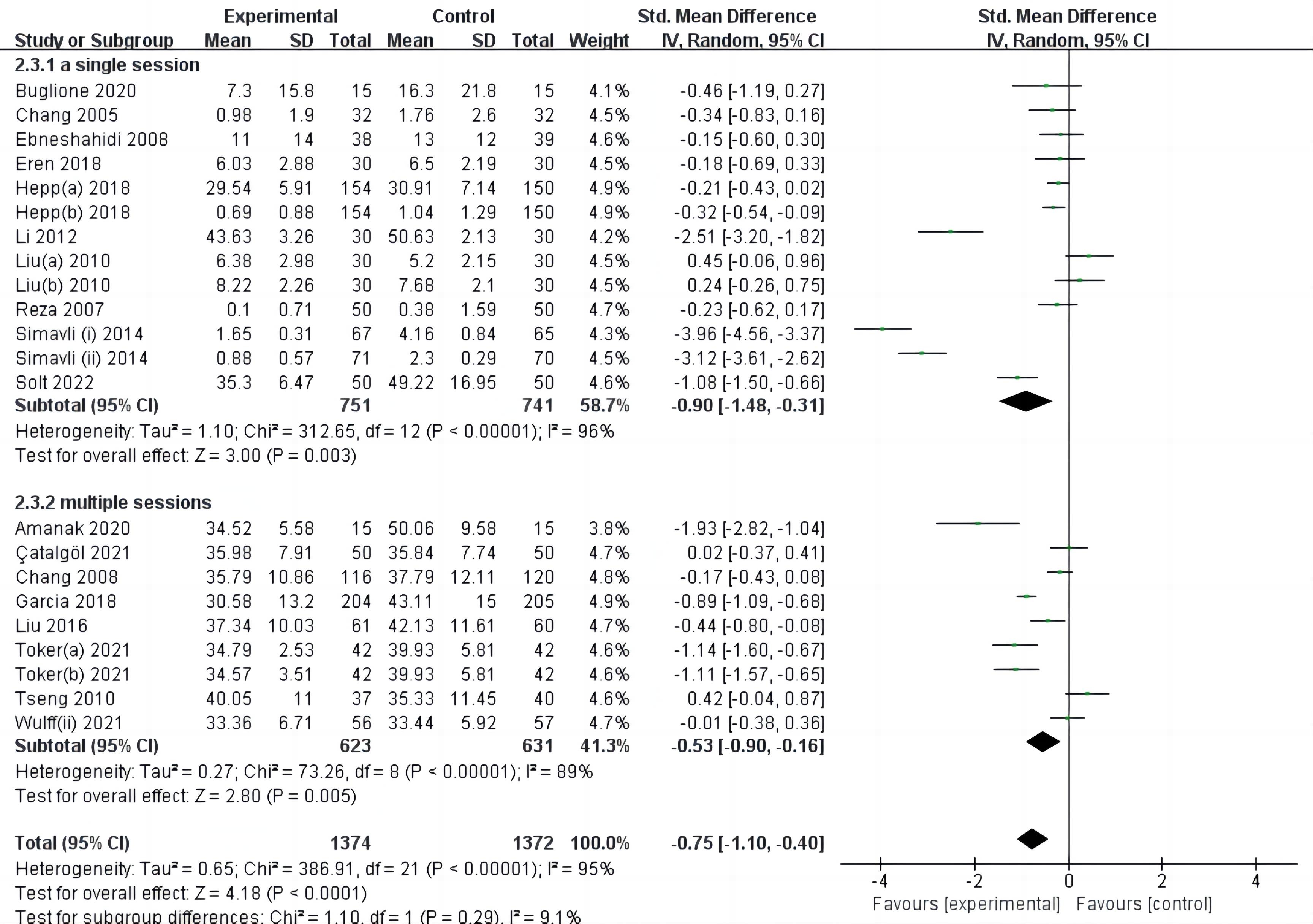

Supplement: Supplementary file 5 [file Image_2.JPEG]

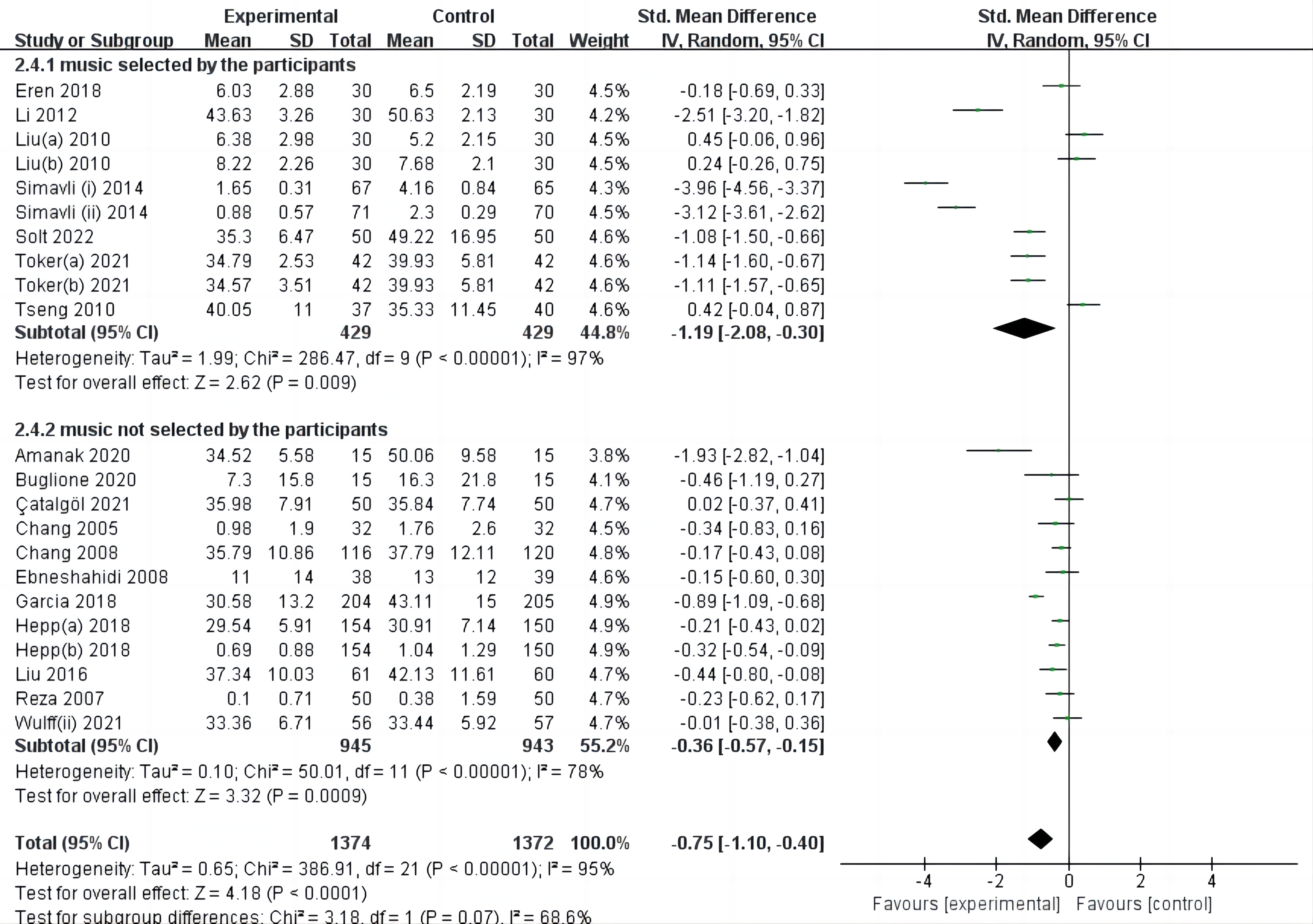

Supplement: Supplementary file 6 [file Image_3.JPEG]
